# Genome-Wide Identification of *Laminin* Family Related to Follicular Pseudoplacenta Development in Black Rockfish (*Sebastes schlegelii*)

**DOI:** 10.3390/ijms231810523

**Published:** 2022-09-10

**Authors:** Ning Zhao, Xueying Wang, Tao Wang, Xiaojie Xu, Qinghua Liu, Jun Li

**Affiliations:** 1 CAS and Shandong Province Key Laboratory of Experimental Marine Biology, Center for Ocean Mega-Science, Institute of Oceanology, Chinese Academy of Sciences, Qingdao 266071, China; 2 Laboratory for Marine Biology and Biotechnology, Qingdao National Laboratory for Marine Science and Technology, Qingdao 266071, China; 3 University of Chinese Academy of Sciences, Beijing 100049, China; 4 School of Marine Science and Engineering, Qingdao Agricultural University, Qingdao 266071, China

**Keywords:** *Sebastes schlegelii*, follicular pseudoplacenta, *laminin* genes, evolution

## Abstract

As major elements of the basement membrane, laminins play a significant role in angiogenesis, migration, and adhesion of various cells. *Sebastes schlegelii* is a marine viviparous teleost of commercial importance. Previous research has reported abundant blood vessels and connective tissue in the ovary during gestation. In this study, 14 *laminin* genes of the α, β, and γ subfamilies from genomic data were identified based on zebrafish and human laminins, distributed on 9 chromosomes in *S. schlegelii*. Analysis of structural domains showed that coiled-coil regions and EGF domains existed in all *laminin* genes. Moreover, via qPCR, we found that the expression of *laminin* genes, including *lama4*, *lama5*, *lamb4*, *lamc1*, and *lamc3*, gradually increased from the phase III ovary stage and peaked in the early stage of gestation, especially *lama4* and *lama5* which showed dramatically increased expression at the blastula stage. Accordingly, in situ hybridization of *lama4* was conducted. The results revealed that signals became stronger following the phase IV ovary stage, and the strongest signals were located on the follicular pseudoplacenta at the blastula stage. These results suggest that the high expression of *laminin* genes, especially *lama4* after fertilization, may drive cell proliferation, migration, and tissue expansion in the *S. schlegelii* ovary and ultimately promote follicular pseudoplacenta formation.

## 1. Introduction

There have been independent origins of viviparity which means that embryos are retained in the female reproductive system or in the body cavity after fertilization and receive nourishment from their yolk sacs or mother in vertebrates [1]. Viviparity is rare in the teleost, with only 13 clades of teleost practising this method of reproduction [2]. In some viviparous bony fish, a critical link between mother and embryo is established through the placenta. A “follicular placenta”, consisting of the belly sac of the embryo, including vastly vascularized pericardial and yolk sacs, and the ovarian vascular follicle wall surrounding the embryo, was first described in studies of the Poeciliidae family [3,4]. Subsequent studies on the structure of the follicular placenta of the *Poeciliopsis* genus showed that both thin and thick maternal follicular walls expressed placenta functional genes [5]. Members of the Goodeidae were found to change their source of nutrition from lecithotrophic (early embryonic development) to matrotrophic nutrition via a branchial placenta and, mainly, trophotaenia [6]. Moreover, histological analysis of the brood pouch of mature male pot-bellied seahorses showed that pseudoplacenta, comprising connective tissue and blood vessels, were located on the innermost side of the pouch and acted as a support for the embryo [7].

The laminin family of glycoproteins are characterized by large molecular weight [8]. Laminin was first isolated and purified from a tumor in lathyritic mice and an epicyte of an embryonic cancer cell line [9,10]. Three chains, one each from α, β, and γ subfamilies, intertwine to form trimers at the laminin coiled-coil region [11,12]. All three laminin subunits contain six domains, such as LamNT and lamEGF. In addition, the α chains contain five lamG domains [13,14]. The C-terminal and N-terminal ends of laminins enable them to interact with molecules on the membrane or in the extracellular matrix [12]. Laminins interact with cells by anchoring to the cellular receptors directly or indirectly. On the one hand, laminins can bind integrin, α-dystroglycan, heparan sulfates, and sulfated glycolipids, which are receptors on the cell surface, with LG domains interacting directly with the cells. On the other hand, laminins can connect with cells circumlocutorily using “bridges”, such as agrin, perlecan, and nidogen, which can link up the laminins and receptors [15].

As major elements of the basement membrane, laminins play an important role in regulating the adhesion, migration, differentiation, and anti-apoptosis of various cells [16,17]. For example, the location and distribution areas of laminin 332 were related to B cell chronic lymphocytic leukemia (B-CLL), since the protein was dispersed in the lymph nodes around the blood vessels of the patient and related to B-CLL cell mobility [18]. Previous reports have shown that laminin 411 secreted by neutrophils acted as a promoter of neutrophil migration with or without chemical stimulation [19]. By interacting with αMβ2-intergrin, laminin 411 accelerated neutrophil passage through albumin-coated filters.

The role of laminin has primarily been studied in stem cells. It was reported that, under treatment with different laminins, such as laminin 211 and laminin 332, human-induced pluripotent stem cells selectively differentiated into divergent eye-like tissues, such as neural crest cells and corneal epithelial cells, by proliferating and migrating outward [20]. Laminin 511 and laminin 521 pluripotent embryonic stem cells were involved in the differentiation of stem cells into other cells, such as cardiac muscle fibers and skin keratinocytes [21]. More specifically, with laminin 521 serving as the stroma layer, human embryonic stem cells survived for more than four months in a single layer and could be identified using Oct4 and Sox-2, which are signs of cellular pluripotency [22].

Apart from the above functions of laminins, the influence of laminin chains, especially *lama4*, *lama5*, *lama1*, *lamb1*, and *lamc1*, in the placenta and embryo development has recently also attracted attention.

Lama4 takes part in the development of the placenta. In humans, lama4 has been detected with tissue-specific expression in the placenta, ovary, skin, heart, lung, skeletal muscle, and pancreas [23,24]. The normal expression of lama4 maintains the stability of placental function. The protein content and gene expression of lama4 in the placenta of pregnant women with preeclampsia are lower than those of normal pregnant women [25]. The elevated expression level of lama4 promotes the migration ability of trophoblast cells. In vitro experiments have confirmed that reducing the expression of lama4 inhibits trophoblast cell migration, which may be related to the occurrence of this disease [26].

In addition, other laminin subunits, such as *lama5*, *lamb1*, and *lamc1*, are necessary during early embryonic development. *Lama5* knockout in mouse embryos affected the connections within the embryonic vascular system and thus influenced the function of the placenta for nutrient and gas transportation, ultimately causing embryo death [27]. Similarly, the mutation of *lamb1* and *lamc1* caused embryo death at E5.5 by hindering laminin formation [28].

The Blackrock fish (*Sebastes schlegelii*), which belongs to the *Sebastes* genus, can be found in the shallow coastal reefs of China, Korea, and Japan [29,30]. As an important fishery resource, it plays an important economic role in the aquatic industry. As a viviparous fish, *S. schlegelii* embryos continue to grow in the ovary of the female fish after fertilization until delivery. Pregnancy usually starts in the middle of April and lasts about one and a half months [31]. In the early stages of embryonic development, the blood vessels and connective tissue begin developing in *S. schlegelii* ovaries [32,33]. The embryos are then surrounded by a “follicular pseudoplacenta”, which includes connective tissues and blood vessels and is linked to the mother via a “follicle stalk”, just as grape flesh is wrapped in the skin [33].

Experiments to explore the function of *laminin* genes have mainly been carried out on mammals, such as humans and mice [12,28]. Previously, most functional validation experiments of *laminin* genes have been carried out in zebrafish [34,35,36,37]; however, fewer studies of *laminin* genes have been performed in viviparous fish. The purpose of this study was to identify the *laminin* genes from the *S. schgelii* genome, analyze the evolution and expression characteristics of possible *laminin* genes related to the follicular pseudoplacenta at different ovarian development stages, and finally to provide information for the study of the reproductive mode of viviparous fish.

## 2. Results

### 2.1. Blackrock Fish Laminin Chains

Zebrafish and human laminin amino acid sequences were employed to screen the *laminin* genes in *S. schlegelii*. We identified 14 laminin subunits in *S. schlegelii*. As shown in Table 1, α chains included five members: *lama1*, *lama2*, *lama3*, *lama4*, and *lama5*; β chains comprised six members: *lamb1a*, *lamb1b*, *lamb2l*, and *lamb2*, *lamb3*, and *lamb4* ; and γ chains included *lamc1*, *lamc2*, and *lamc3*. We forecast specific physicochemical properties according to the basal amino acids. The amino acids of the laminin subunits varied from 622 (*lamb4*) to 3666 (*lama5*), with molecular weights ranging from 70.517 to 401.73. The theoretical PI of laminin chains was lower than 7.0, except for laminin α3. Excluding laminin α 1, 3, and 4, and laminin β 2l located on the plasma membrane, a significant proportion of the laminin chains were predicted to be secreted proteins distributed outside the cell.

### 2.2. Phylogenic Analysis of Laminin Chains

**Figure 1:**
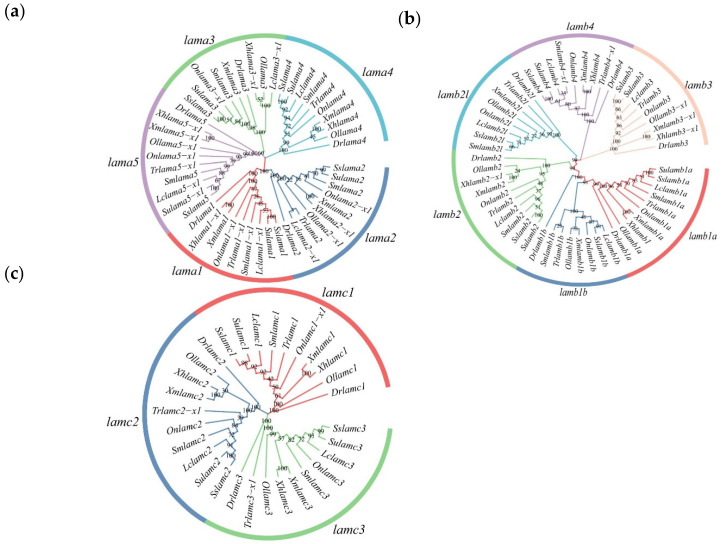
Phylogenetic analysis of laminin nucleotide sequence of ten species. Dr (*Danio rerio*), Lc (*Larimichthys crocea*), Sm (*Scophthalmus maximus*), Ol (*Oryzias latipes*), Tr (*Takifugu rubripes*), On (*Oreochromis niloticus*), Xm (*Xiphophorus maculatus*), Xh (*Xiphophorus helleri*), Ss (*S. schlegelii*) and Su (*S. umbrosus*). The numbers on the graph represent approval ratings. (**a**) Phylogenetic tree of laminin α chains; (**b**) Phylogenetic tree of laminin β chains; (**c**) Phylogenetic tree of laminin γ chains. The same gene from different species comes together to form a single branch with the same color.

Phylogenic trees of *S. schlegelii* and nine other fish laminin α, β, and γ chains were constructed to confirm the laminin chains in *S. schlegelii* (Figure 1). The number and type of laminin chains were conserved in oviparous and viviparous fish. The laminin α chains contained five clades, while the β and γ chains had six and three branches, respectively. The laminin chains of *S. schlegelii* always clustered with *Sebastes umbrosus* belonging to the same genus (*Sebastes*).

### 2.3. Analysis of Gene Location in S. schlegelii and Other Teleosts

**Figure 2:**
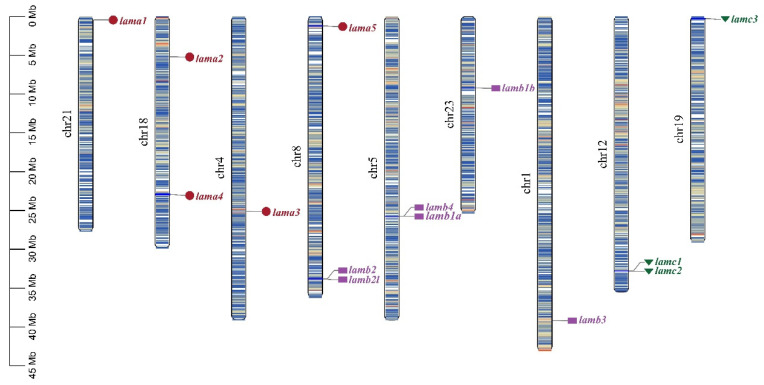
Chromosome location of laminin genes in *S. schlegelii*. Genes of the same subfamily are marked by the same color and shade. Chr: chromosome.

**Figure 3:**
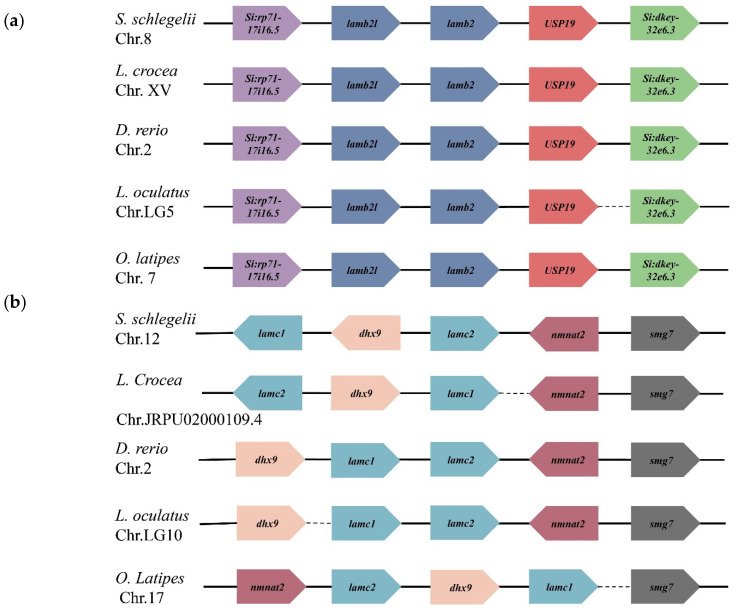
The position of tandem laminin genes in teleosts. The arrow direction indicates the direction of transcription. Dashed lines indicate discontinuities between genes. Genes of the same gene or family are represented in the same color. (**a**) Orders of *lamb2* and *lamb2l* in teleosts; (**b**) Orders of *lamc1* and *lamc2* in teleosts.

The location of *laminin* genes on chromosomes was determined based on a genome database. As shown in Figure 2, 15 *laminin* genes were distributed on nine chromosomes. Laminin α2 and 4 chains were both located on chromosome 18; moreover, four other pairs of genes (as shown in Figure 2) were also located on the same chromosome. To clearly display the *laminin* genes, which were next to each other on the same chromosome, we present a location schematic plot of *lamb2l*, *lamb2*, *lamc1*, *lamc2* (Figure 3) in teleosts. The transcription directions and upstream and downstream genes of *lamb2* and *lamb2l* were relatively consistent in teleosts. *Lamc1* and *lamc2* were positioned in tandem or separated by *dhx9*. Furthermore, the transcription direction of *lamc1* was identical with *dhx9*. However, although *lamb4* and *lamb1a* were arranged in tandem on the same chromosome, the genes next to them were not identical in different fish. 

### 2.4. Gene Structure and Conserved Domain

**Figure 4:**
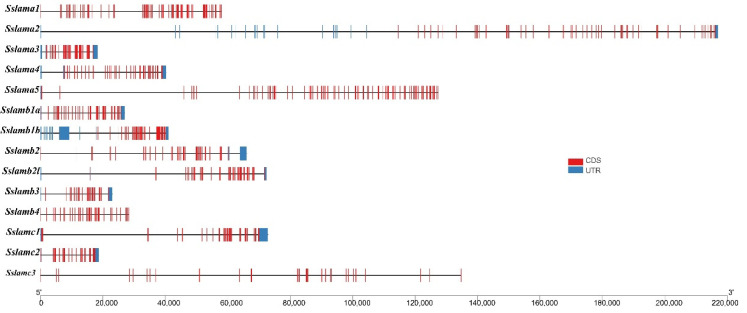
Gene structures of 14 *laminin* genes. The black solid line represents the intron region. The blue rectangle represents the UTR region, and the red rectangle represents the exon region.

**Figure 5:**
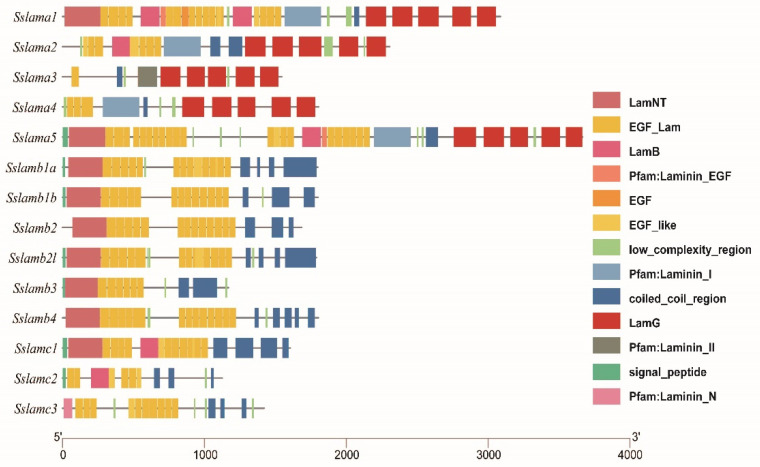
Conserved domains analysis of laminin genes. The number represents the length of the amino acid. Different colors of rectangle represent diverse domains.

To better understand the structure of *laminin* genes, we drew schemes of the gene structure and conserved domain of *laminin* genes, as shown in Figure 4 and Figure 5. Laminin chains with long mRNA sequences were predicted to have relatively scattered exons. Coiled-coil and EGF domains were present in all *laminin* genes. Five LamG domains appeared in laminin α chains. Signal peptide domains existed in laminin β and γ chains, which was consistent with their subcellular location.

### 2.5. Laminin Gene Expression Analysis at Different Ovarian Stages 

**Figure 6:**
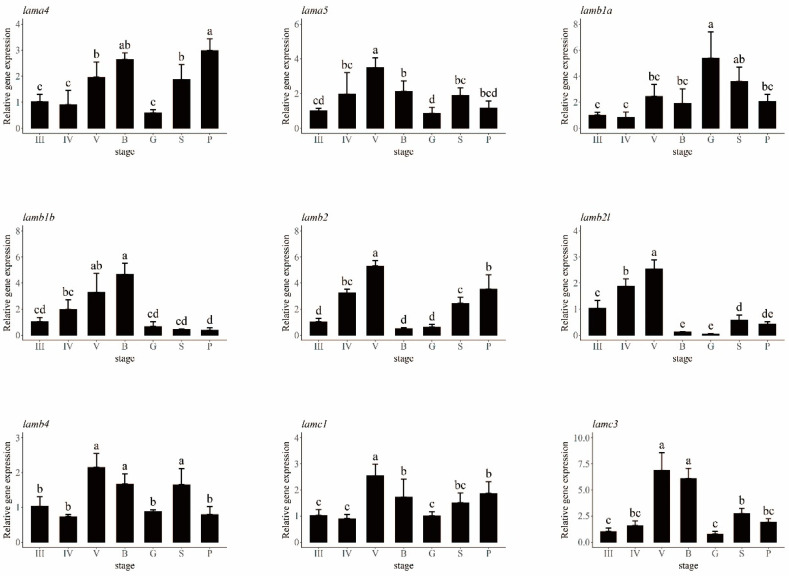
Expression analysis of laminin genes at seven different ovarian development stages. The relative expression levels of laminin genes are shown in different colors. III: ovary at stage III; IV: ovary at stage IV; V: ovary at stage V; B: blastula stage; G: gastrula stage; S: somites stage; P: prehatching stage. Different letters are signs of significant differences.

The ovaries of *S. schlegelii* in eight different stages—II, III, IV, V, blastula, gastrula, somite, and pre-hatching—were used to obtain the relative expression of *laminin* genes using quantitative PCR (qPCR) (Figure 6). Different *laminin* genes did not follow the same pattern. The laminin α3 gene was not expressed in *S. schlegelii* ovaries, according to the qPCR results. The expression of the laminin α2 gene stayed at a similar level in different stages with no significant differences noted (Figure S1). *Lamb3* and *lamc2* were expressed at low levels until the pre-hatching stage, which indicates that these two genes may work mainly in the juvenile stage. The relative expression levels of other genes tended to take a parabolic shape. Moreover, *lama4*, *lama5*, *lamb1b*, *lamb2l*, *lamb2*, *lamb4*, and *lamc1* and *lamc3* seemed to participate in follicular pseudoplacenta formation, because they were expressed at significantly higher levels at stage V, or the blastula stage, when the follicular placenta became most vigorous. *Lamb1b* expression levels lessened from the blastula to the gastrula stage, while *lamb1a* showed the opposite trend. *Lamb2l* and *b2* gradually increased from stage III to V and significantly lessened from stage V to the blastula stage.

### 2.6. The Location of Lama4 in Reproductive Cycle of S. schlegelii

**Figure 7:**
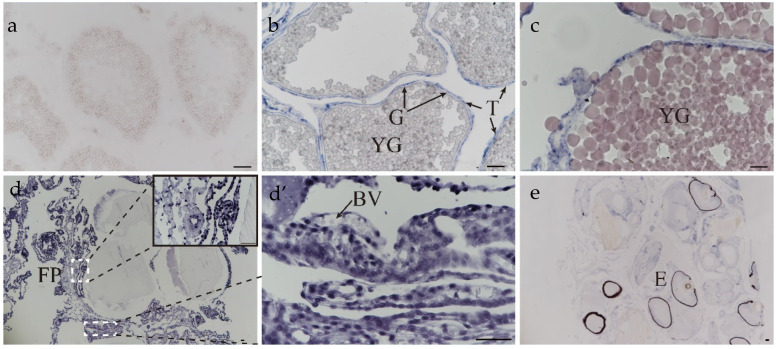
The location of lama4 in ovaries during oogenesis and gestation. (**a**) ovary at stage III; (**b**) ovary at stage IV—YG: yolk globule; T: theca cell; G: granulosa cell; (**c**) ovary at stage V; (**d**) embryos at the blastula stage—B: blastula stage, FP: follicular pseudoplacenta; (**d’**) follicular pseudoplacenta at the blastula stage—BV: blood vessel; (**e**) embryos at the prehatching stage—E: embryo. scale bar: 50 μm.

An experiment to detect the location of *lama4* was conducted. The variety of *lama4* in the ovary at different stages is shown in Figure 7. No signal for *lama4* was detected in the ovary at the III stage (Figure 7a). Signals became stronger during oocyte development and pregnancy, being strongest at the blastula stage (Figure 7b–d’). *Lama4* was first detected on the theca cells at the IV stage ovary (Figure 7b); there was no positive signal detected on granulosa cells and oocytes at this point. At the blastula stage, intense responses to the *lama4* probe were produced on the follicular pseudoplacenta and egg membranes (Figure 7d,d’). Before delivery, *lama4* was expressed at a low level and was present on the follicular pseudoplacenta, egg membranes, and embryos (Figure 7e).

## 3. Discussion

The number of laminin genes has gradually increased during evolution, from two chains in Radiata to 11 chains in vertebrates; apart from the anole lizard and frog, vertebrates have multiple α, β and γ chains [38]. There are 11 and 12 laminin genes in the mouse and human, respectively. The third genome duplication in fish evolution led to new laminin chains in teleosts [38,39]. Compared with other vertebrates, the extra laminin genes in teleosts are mainly *lamb1a*, *lamb1b*, *lamb2* and *lamb2l*. In this study, we identified 14 members in the *S. schlegelii* genome—*lama1*, *lama2*, *lama3*, *lama4*, *lama5*, *lamb1a*, *lamb1b*, *lamb2l*, *lamb2*, *lamb3*, *lamb4*, *lamc1*, *lamc2*, and *lamc3*—which were consistent with the laminin genes found in zebrafish. There was no expansion or contraction of the laminin family compared to other teleosts. Phylogenetic analysis showed that 10 species of the same gene clustered on one branch, indicating that the laminin gene is highly conserved among teleosts. Gene localization on chromosomes revealed the irregular distribution of laminin genes on chromosomes, but tandem distribution of genes appeared on chromosomes 5 and 8. Tandem genes showed a high degree of consistency in the order of arrangement and transcriptional direction on chromosomes in teleosts.

The members of the laminin gene family are divided into three subfamilies: α, β and γ. Laminin EGF and coiled-coil domains are present in all laminin genes. Three chains assemble at the coiled-coil domain to form a trimer of laminin proteins. Five LamG domains are unique to the α chains. Laminins participate in the cell adhesion process by binding to integrins via the LamG domains [40,41].

The qPCR results showed that highly expressed genes in phase V, or the blastula stage, included *lama4*, *lama5*, *lamb4*, *lamc1*, and *lamc3*. *lama4* was expressed at a dramatically high level at phase V and the blastula stage. By conducting ISH of *lama4*, we found that signals became stronger during oocyte development and pregnancy, being the strongest at the blastula stage, consistent with the qPCR results. Similarly, laminin staining of female rat ovaries became stronger in the basement membrane and granulosa cells until the Graafian follicle stage [42]. A previous study reported that lama4 was localized in the human placenta and strongly expressed in early gestation. The decrease of *lama4* expression influenced angiogenesis and the invasion and migration of trophoblasts [43]. This suggests that *lama4* in *S.schlegelii* may accelerate the invasion and migration of cells to form follicular pseudoplacenta and maintain normal maternal-embryonic contact.

In our analysis, *lama3* was not detected in *S. schlegelii* ovaries. This result is consistent with previous research [44,45]. Analysis of *lama3* expression in mouse tissues found that *lama3* was expressed highly in the lung and skin, where epithelial cells represent a large proportion of cells. Furthermore, RNase protection results revealed no *lama3* expression in the placenta [45]. In vitro, the repression of *lama3* gene expression is also cell-specific. For instance, *lama3* was not expressed in fibroblasts; conversely, expression remained at a high level in keratinocytes [44]. During the ovarian development of adult female *S. schlegelii*, *lamb1b* expression slowly increased to its maximum in the blastula stage, rapidly decreased in the gastrula stage, and stayed at a low level until delivery; however, *lamb1a* decreased slowly and was maintained at a high level. We suggest that lamb1a and lamb1b may alternately participate in connective tissue expansion.

## 4. Materials and Methods

### 4.1. Identification of Laminin Genes

To identify possible laminin subunits in *S. schlegelii*, zebrafish and humans, laminin protein sequences obtained from NCBI (https://www.ncbi.nlm.nih.gov/, accessed on 25 November 2021) and using the Ensembl Genome Browser at http://www.ensembl.org/ and Hidden Markov (accessed on 25 November 2021) Model of laminin family downloaded at Pfam 35.0 (http://pfam.xfam.org/, accessed on 26 June 2022) were used as query sequences against the *S. schlegelii* genome database in TBtools (E-value < 1 × 10^−5^ [46]. The accession numbers and pfam id previously mentioned are shown in Table S1. The first BLAST hits were then conserved and used as BLATP query sequences in the UniProtKB/Swiss-Prot (swissprot) database. Finally, potential laminin sequences were certified according to their annotation and conserved domains.

### 4.2. Gene Locations, Conserved Domains, Gene Structure, and Physicochemical Properties

*Laminin* gene locations in *S. schlegelii* and other teleosts were predicted using Genomicus [Genomicus v106.01-Gene Search (psl.eu)] [47]. Conserved domains were predicted via SMART (http://smart.embl-heidelberg.de/, accessed on 31 July 2022) and redrawn using TBtools. Gene structures were visualized using TBtools. The molecular weight and theoretical PI of the laminin proteins were analyzed using ProtParam (http://web.expasy.org/protparam/, accessed on 31 July 2022). The subcellular localization information of the laminin proteins was predicted via ProtComp (http://linux1.softberry.com/, accessed on 31 July 2022).

### 4.3. Phylogenetic Analysis of Laminin Genes

Laminin-coding DNA sequences of nine fish (*Danio rerio*, *Larimichthys crocea*, *Scophthalmus maximus*, *Oryzias latipes*, *Takifugu rubripes*, *Oreochromis niloticus*, *Xiphophorus maculatus*, *Xiphophorus helleri*, and *S. umbrosus*) were downloaded from NCBI (https://www.ncbi.nlm.nih.gov/, accessed on 29 December 2021) and using the Ensembl Genome Browser (http://www.ensembl.org/, accessed on 29 December 2022). The accession numbers of nucleic acid sequences are shown in Table S2. Coding sequences were aligned in the align codons model using MEGA 11 software. NJtree was constructed using MEGA11 software, and the reliability was assessed using bootstrap analysis set at 1000 times.

### 4.4. RNA Extraction and qPCR Analysis of Gene Expression

Adult female fish were bought from Nanshan market (Qingdao, Shandong Province, China) every other month or two weeks during the ovary development stage and anesthetized using MS-222 (300 mg/mL). The gonads were collected in triplicate from each fish. Samples used in situ were fixed overnight in 4% paraformaldehyde and then stored at −20 °C in 70% alcohol; other samples were stored in liquid nitrogen at once and used later for total RNA extraction.

Total RNA was isolated using TRIzol according to the manufacturer’s instructions. The purity and concentration of extracted RNA was tested using a NanoDrop 2000 spectrophotometer (Termo Scientifc, Shanghai, China), and the degree of RNA degradation was examined using gel electrophoresis. Reverse transcription was carried out according to the operation manual of the Evo M-MLV RT Mix kit with gDNA Clean for qPCR (Accurate Biology). The specific primers of *laminin* genes were designed using Primer Premier 5 (Table S1). QPCR using SYBR Green Premix Pro Taq HS qPCR Kit (Accurate Biology) was performed using a Bio-Rad CFX Connect™ Real-Time PCR System (Bio-Rad, Hercules, CA, USA). The 18 s gene was regarded as a reference, and each reaction was performed in three to six independent biological replicates. The program was set as follows: 95 °C for 3 min, followed by 39 cycles at 95 °C for 10 s, 59 °C for 30 s, and 72 °C for 30 s. Finally, the relative expression of each *laminin* gene was calculated using the 2^−ΔΔCT^ method and the significance between different stages was tested by one-way ANOVA and Duncan’s Multiple Range Test in R (version 4.2.0).

### 4.5. In Situ Hybridization

The primers of the *lama4* probe are listed in Table S3 [32]. RNA probes of *lama4* were then synthesized using the DIG RNA Labeling Kit (Roche, Mannheim, Germany), purified on SigmaSpin^TM^ sequencing reaction clean-up columns (Sigma-Aldrich, St. Louis, MO, USA), and stored at −80 °C. Slices of 7 μm thickness of gonads were obtained using a Leica RM2235 microtome after dehydration and embedded. In situ hybridization experiments were performed according to the method described by Wang et al. [48].

## 5. Conclusions

We identified 14 laminin chains of the α, β, and γ subfamilies and analyzed their evolutionary relationships, and the expression patterns in teleosts and in the ovaries of *S. schlegelii*. We also found that laminin genes, especially lama4, which are strongly expressed in the early stages of pregnancy in *S. schlegelii*, may be related to the emergence of the follicular pseudoplacenta. The results can support laminin studies in teleost fish, especially viviparous fish. More importantly, they can help to determine the role of laminin genes in the formation of the follicular pseudoplacenta.

## Figures and Tables

**Table 1:** Physicochemical properties of laminin.

Gene	Number of Amino Acids	Molecular Weight (kDa)	Theoretical pI	Subcellular Location
*lama1*	3087	333.5	5.73	Membrane bound Extracellular (Secreted)
*lama2*	2306	251.46	6.38	Membrane bound Extracellular (Secreted)
*lama3*	1544	171.9	7.15	Plasma membrane
*lama4*	1804	200.16	5.6	Plasma membrane
*lama5*	3666	401.73	5.64	Membrane bound Extracellular (Secreted)
*lamb1a*	1799	197.73	4.99	Membrane bound Extracellular (Secreted)
*lamb1b*	1800	197.81	4.94	Membrane bound Extracellular (Secreted)
*lamb2l*	1789	199.48	6.27	Plasma membrane
*lamb2*	1685	184.97	6.31	Membrane bound Extracellular (Secreted)
*lamb3*	1169	128.05	5.37	Extracellular (Secreted)
*lamb4*	1803	199.87	5.34	Membrane bound Extracellular (Secreted)
*lamc1*	1605	176.31	5.16	Membrane bound Extracellular (Secreted)
*lamc2*	1125	121.23	5.95	Membrane bound Extracellular (Secreted)
*lamc3*	1421	156.92	5.72	Membrane bound Extracellular (Secreted)

## Data Availability

Not applicable.

## Supplementary Materials

The supporting information can be downloaded at: https://www.mdpi.com/article/10.3390/ijms231810523/s1.

## References

[1] Wourms J.P. (1981). Viviparity: The Maternal-Fetal Relationship in Fishes. Am. Zool..

[2] Blackburn D.G. (2015). Evolution of vertebrate viviparity and specializations for fetal nutrition: A quantitative and qualitative analysis. J. Morphol..

[3] Turner C.L. (1940). Pseudoamnion, pseudochorion, and follicular pseudoplacenta in poeciliid fishes. J. Morphol..

[4] Grove B.D., Wourms J.P. (1994). Follicular Placenta of the Viviparous Fish, *Heterandria formosa*: II. Ultrastructure and Development of the Follicular Epithelium. J. Morphol..

[5] Guernsey M.W., van Kruistum H., Reznick D.N., Pollux B.J.A., Baker J.C. (2020). Molecular Signatures of Placentation and Secretion Uncovered in *Poeciliopsis* Maternal Follicles. Mol. Biol. Evol..

[6] Uribe M.C., Grier H.J., Avila-Zuniga S.A., Garcia-Alarcon A. (2018). Change of lecithotrophic to matrotrophic nutrition during gestation in the viviparous teleost *Xenotoca eiseni* (Goodeidae). J. Morphol..

[7] Kawaguchi M., Okubo R., Harada A., Miyasaka K., Takada K., Hiroi J., Yasumasu S. (2017). Morphology of brood pouch formation in the pot-bellied seahorse *Hippocampus abdominalis*. Zool. Lett..

[8] Sasaki M., Kleinman H.K., Huber H., Deutzmann R., Yamada Y. (1988). Laminin, a multidomain protein. The A chain has a unique globular domain and homology with the basement membrane proteoglycan and the laminin B chains. J. Biol. Chem..

[9] Timpl R., Rohde H., Robey P.G., Rennard S.I., Foidart J.M., Martin G.R. (1979). Laminin–a glycoprotein from basement membranes. J. Biol. Chem..

[10] Chung A.E., Jaffe R., Freeman I.L., Vergnes J.P., Braginski J.E., Carlin B. (1979). Properties of a basement membrane-related glycoprotein synthesized in culture by a mouse-embryonal carcinoma-derived cell-line. Cell.

[11] Malinda K.M., Kleinman H.K. (1996). The laminins. Int. J. Biochem. Cell Biol..

[12] Aumailley M. (2013). The laminin family. Cell Adh. Migr..

[13] Fahey B., Degnan B.M. (2012). Origin and evolution of laminin gene family diversity. Mol. Biol. Evol..

[14] Beck K., Hunter I., Engel J. (1990). Structure and function of laminin-anatomy of a multidomain glycoprotein. FASEB J..

[15] Hohenester E., Yurchenco P.D. (2013). Laminins in basement membrane assembly. Cell Adh. Migr..

[16] Maltseva D.V., Rodin S.A. (2018). Laminins in Metastatic Cancer. Mol. Biol..

[17] Eble J.A., Niland S. (2009). The Extracellular Matrix of Blood Vessels. Curr. Pharm. Des..

[18] Spessotto P., Zucchetto A., Degan M., Wasserman B., Danussi C., Bomben R., Perris R., Canzonieri V., Radillo O., Colombatti A. (2007). Laminin-332 (Laminin-5) is the major motility ligand for B cell chronic lymphocytic leukemia. Matrix Biol..

[19] Wondimu Z., Geberhiwot T., Ingerpuu S., Juronen E., Xie X., Lindbom L., Doi M., Kortesmaa J., Thyboll J., Tryggvason K. (2004). An endothelial laminin isoform, laminin 8 (alpha4beta1gamma1), is secreted by blood neutrophils, promotes neutrophil migration and extravasation, and protects neutrophils from apoptosis. Blood.

[20] Shibata S., Hayashi R., Okubo T., Kudo Y., Katayama T., Ishikawa Y., Toga J., Yagi E., Honma Y., Quantock A.J. (2018). Selective Laminin-Directed Differentiation of Human Induced Pluripotent Stem Cells into Distinct Ocular Lineages. Cell Rep..

[21] Yap L., Tay H.G., Nguyen M.T.X., Tjin M.S., Tryggvason K. (2019). Laminins in Cellular Differentiation. Trends Cell Biol..

[22] Rodin S., Antonsson L., Niaudet C., Simonson O.E., Salmela E., Hansson E.M., Domogatskaya A., Xiao Z., Damdimopoulou P., Sheikhi M. (2014). Clonal culturing of human embryonic stem cells on laminin-521/E-cadherin matrix in defined and xeno-free environment. Nat. Commun..

[23] Iivanainen A., Sainio K., Sariola H., Tryggvason K. (1995). Primary structure and expression of a novel human laminin a4 chain. FEBS Lett..

[24] Richards A., Al-lmara L., Pope F.M. (1996). The complete cDNA sequence of laminin a4 and its relationship to the other human laminin a chains. Eur. J. Biochem..

[25] Liu M. (2017). Expression level of LAMA4 protein in placental tissue of preeclampsia patients and its correlation with invasion of trophoblast cells and placental hypoxia. Matern. Child Health Care China.

[26] Wang P., Nan S., Wang Y., Qi H. (2016). Expression and function of LAMA4 in the placentas and trophoblast. J. Chongqing Med. Univ..

[27] Miner J.H., Cunningham J., Sanes J.R. (1998). Roles for laminin in embryogenesis: Exencephaly, syndactyly, and placentopathy in mice lacking the laminin alpha 5 chain. J. Cell Biol..

[28] Miner J.H. (2008). Laminins and their roles in mammals. Microsc. Res. Tech..

[29] Nakagawa M., Okouchi H., Adachi J., Hattori K., Yamashita Y. (2007). Effectiveness of stock enhancement of hatchery-released black rockfish *Sebastes schlegeli* in Yamada Bay—Evaluation by a Fish Market survey. Aquaculture.

[30] Boehlert G.W., Kusakari M., Shimizu M., Yamada J. (1986). Energetics during embryonic-development in Kurosoi, *Sebastes schlegeli* hilgendorf. J. Exp. Mar. Biol. Ecol..

[31] Yamada J., Kusakari M. (1991). Staging and the time course of embryonic-development in Kurosoi, *Sebastes schlegeli*. Environ. Biol. Fishes.

[32] Xu X., Wang X., Liu Q., Qi X., Zhou L., Liu H., Li J. (2022). New insights on folliculogenesis and follicular placentation in marine viviparous fish black rockfish (*Sebastes schlegelii*). Gene.

[33] Tengfei D., Yongshuang X., Haixia Z., Li Z., Qinghua L., Xueying W., Jun L., Shihong X., Yanfeng W., Jiachen Y. (2021). Multiple Fetal Nutritional Patterns Before Parturition in Viviparous Fish *Sebastes schlegelii* (Hilgendorf, 1880). Front. Mar. Sci..

[34] Zinkevich N.S., Bosenko D.V., Link B.A., Semina E.V. (2006). laminin alpha 1 gene is essential for normal lens development in zebrafish. BMC Dev. Biol..

[35] Sittaramane V., Sawant A., Wolman M.A., Maves L., Halloran M.C., Chandrasekhar A. (2009). The cell adhesion molecule Tag1, transmembrane protein Stbm/Vangl2, and Lamininalpha1 exhibit genetic interactions during migration of facial branchiomotor neurons in zebrafish. Dev. Biol..

[36] Paulus J.D., Halloran M.C. (2006). Zebrafish bashful/laminin-alpha 1 mutants exhibit multiple axon guidance defects. Dev. Dyn..

[37] Parsons M.J., Pollard S.M., Saude L., Feldman B., Coutinho P., Hirst E.M.A., Stemple D.L. (2002). Zebrafish mutants identify an essential role for laminins in notochord formation. Development.

[38] Domogatskaya A., Rodin S., Tryggvason K. (2012). Functional diversity of laminins. Annu. Rev. Cell Dev. Biol..

[39] Meyer A., Van de Peer Y. (2005). From 2R to 3R: Evidence for a fish-specific genome duplication (FSGD). Bioessays.

[40] Nielsen P.K., Yamada Y. (2001). Identification of cell-binding sites on the Laminin alpha 5 N-terminal domain by site-directed mutagenesis. J. Biol. Chem..

[41] Sasaki T., Timpl R. (2001). Domain IVa of laminin alpha 5 chain is cell-adhesive and binds beta 1 and alpha V beta 3 integrins through Arg-Gly-Asp. FEBS Lett..

[42] Akkoyunlu G., Demir R., Ustunel I. (2003). Distribution patterns of TGF-alpha, laminin and fibronectin and their relationship with folliculogenesis in rat ovary. Acta Histochem..

[43] Shan N., Zhang X., Xiao X., Zhang H., Tong C., Luo X., Chen Y., Liu X., Yin N., Deng Q. (2015). Laminin alpha4 (LAMA4) expression promotes trophoblast cell invasion, migration, and angiogenesis, and is lowered in preeclamptic placentas. Placenta.

[44] Virolle T., Coraux C., Ferrigno O., Cailleteau L., Ortonne J.P., Pognonec P., Aberdam D. (2002). Binding of USF to a non-canonical E-box following stress results in a cell-specific derepression of the lama3 gene. Nucleic Acids Res..

[45] Miner J.H., Patton B.L., Lentz S.I., Gilbert D.J., Jenkins N.A., Copeland N.G., Sanes J.R. (1997). The laminin alpha chains: Expression, developmental transitions, and chromosomal locations of alpha 1-5, identification of heterotrimeric laminins 8-11, and cloning of a novel alpha 3 isoform. J. Cell Biol..

[46] Chen C., Chen H., Zhang Y., Thomas H.R., Frank M.H., He Y., Xia R. (2020). TBtools: An Integrative Toolkit Developed for Interactive Analyses of Big Biological Data. Mol. Plant..

[47] Nguyen N.T.T., Vincens P., Roest Crollius H., Louis A. (2018). Genomicus 2018: Karyotype evolutionary trees and on-the-fly synteny computing. Nucleic Acids Res..

[48] Wang S., Zhang J., Jiao W., Li J., Xun X., Sun Y., Guo X., Huan P., Dong B., Zhang L. (2017). Scallop genome provides insights into evolution of bilaterian karyotype and development. Nat. Ecol. Evol..

