# Supplementary material for: Genome-Wide Identification of Laminin Family Related to Follicular Pseudoplacenta Development in Black Rockfish (Sebastes schlegelii)
Source: Int J Mol Sci. 2022 Sep 10;23(18):10523. doi: 10.3390/ijms231810523 (PMC9504374; doi:10.3390/ijms231810523)
Supplement: Supplementary file 1 [file ijms-23-10523-s001.zip › Table S1.pdf]

**Protein Name**	**Accession Number**
Drlama1	ENSDARP00000135371
Drlama2	AER12034
Drlama3	ENSDARP00000150169
Drlama4	AAI63717
Drlama5	ENSDARP00000045125
Drlamb1	AAM61767
Drlamb2	ENSDARP00000123269
Drlamb3	XP_700808
Drlamb4	ENSDARP00000058399
Drlamc1	ENSDARP00000024860
Drlamc2	ENSDARP00000089477
Drlamc3	ENSDARP00000117247
Hslama1	ENSP00000374309
Hslama2	ENSP00000400365
Hslama3	ENSP00000324532
Hslama4	ENSP00000230538
Hslama5	ENSP00000252999
Hslamb1	ENSP00000222399
Hslamb2	ENSP00000307156
Hslamb3	ENSP00000348384
Hslamb4	ENSP00000205386
Hslamc1	ENSP00000258341
Hslamc2	ENSP00000264144
Hslamc3	ENSP00000354360
Laminin Family Pfam ID	
PF00052.21	
PF00053.27	
PF00054.26	
PF06008.17	
PF06009.15	
PF00055.20	
