# Supplementary material for: Genome-Wide Identification of Laminin Family Related to Follicular Pseudoplacenta Development in Black Rockfish (Sebastes schlegelii)
Source: Int J Mol Sci. 2022 Sep 10;23(18):10523. doi: 10.3390/ijms231810523 (PMC9504374; doi:10.3390/ijms231810523)
Supplement: Supplementary file 1 [file ijms-23-10523-s001.zip › Table S2.pdf]

**Gene Name**	**Accession Number**
Drlama1	NM_001034986
Drlama2	NM_001278799
Drlama3	XM_005161726
Drlama4	NM_001039065
Drlama5	NM_001039171
Drlamb1a	ENSDART00000170673
Drlamb1b	ENSDART00000066945
Drlamb2	ENSDART00000147326
Drlamb2l	ENSDART00000048366
Drlamb3	XM_695716
Drlamb4	NM_173276
Drlamc1	NM_173277
Drlamc2	XM_003197884
Drlamc3	XM_682251
Lclama1	XM_019258768
Lclama2	XM_019261641
Lclama3	XM_019262254
Lclama4	XM_010735896
Lclama5	XM_027288364
Lclamb1a	ENSLCRT00005015349
Lclamb1b	ENSLCRT00005051845
Lclamb2l	ENSLCRT00005066025
Lclamb2	ENSLCRT00005065676
Lclamb3	XM_010735318
Lclamb4	XM_019257787
Lclamc1	XM_027275905
Lclamc2	XM_010753625
Lclamc3	XM_019269141
Onlama1	XM_005460467
Onlama2	XM_019345948
Onlama3	XM_025903152
Onlama4	XM_005457921
Onlama5	XM_013271787
Onlamb1a	ENSONIT00000059121
Onlamb1b	ENSONIT00000022528
Onlamb2l	ENSONIT00000002002
Onlamb2	ENSONIT00000001983
Onlamb3	XM_025905132
Onlamb4	XM_019361994
Onlamc1	XM_003458942
Onlamc2	XM_003458940
Onlamc3	XM_005454536

Gene Name	Accession Number
Ollama2	XM_023952768
Ollama3	XM_023964954
Ollama4	XM_023952973
Ollama5	XM_023957048
Ollamb1a	ENSORLT00000000448
Ollamb1b	ENSORLT00000020952
Ollamb2	ENSORLT00000041438
Ollamb2l	ENSORLT00000038914
Ollamb3	XM_011475210
Ollamc1	NM_001201523
Ollamc2	XM_011486435
Ollamb2	ENSORLT00000028933
Ollamc3	ENSORLT00000027901
Smlama1	XM_035619642
Smlama2	XM_035616735
Smlama5	XM_035643819
Smlamb1a	XM_035642935
Smlamb1b	XM_035610822
Smlamb2l	ENSSMAT00000006538
Smlamb2	ENSSMAT00000076043
Smlamb4	XM_035643424
Smlamc1	XM_035635552
Smlama3	ENSSMAT00000052599
Smlama4	ENSSMAT00000028462
Smlamc2	ENSSMAT00000071114
Smlamc3	ENSSMAT00000017383
Sulama1	XM_037763358
Sulama2	XM_037750093
Sulama3	XM_037770230
Sulama4	XM_037750776
Sulama5	XM_037773183
Sulamb1a	XM_037768124
Sulamb2	XM_037772241
Sulamb3	XM_037764858
Sulamb4	XM_037768708
Sulamc1	XM_037787337
Sulamc2	XM_037787365
Sulamc3	XM_037753305
Trlamc1	XM_029830428
Trlama2	XM_029850012
Trlamc3	XM_029837049
Trlama1	XM_029842712

Gene Name	Accession Number
Trlamc2	XM_029831511
Trlamb2l	ENSTRUT00000063997
Trlamb2	ENSTRUT00000061525
Trlama4	XM_011618960
Trlamb3	XM_011613631
Trlamb4	XM_029831844
Trlamb1b	ENSTRUT00000038905
Trlamb1a	ENSTRUT00000076136
Trlama5	XM_029834335
Xhlama1	XM_032551424
Xhlama2	XM_032584629
Xhlama3	XM_032564924
Xhlama4	XM_032584535
Xhlama5	XM_032560267
Xhlamb1	XM_032588660
Xhlamb2	XM_032568959
Xhlamb3	XM_032550055
Xhlamb4	XM_032549289
Xhlamc1	XM_032564369
Xhlamc2	XM_032564395
Xhlamc3	XM_032570011
Xmlama1	XM_023326834
Xmlama2	XM_023347282
Xmlama3	XM_023340088
Xmlama4	XM_014471392
Xmlama5	XM_005801930
Xmlamb1a	ENSXMAT00000016267
Xmlamb1b	ENSXMAT00000004475
Xmlamb2	ENSXMAT00000016566
Xmlamb2l	ENSXMAT00000016576
Xmlamb3	XM_014474650
Xmlamb4	XM_014473775
Xmlamc1	XM_023335263
Xmlamc2	XM_023335281
Xmlamc3	XM_014468646
