# Supplementary material for: Genome-Wide Identification of Laminin Family Related to Follicular Pseudoplacenta Development in Black Rockfish (Sebastes schlegelii)
Source: Int J Mol Sci. 2022 Sep 10;23(18):10523. doi: 10.3390/ijms231810523 (PMC9504374; doi:10.3390/ijms231810523)
Supplement: Supplementary file 1 [file ijms-23-10523-s001.zip › Table S3.pdf]

Laminin gene name	Primer sequences (5'-3')	purpose
Lama1	GGACGTGGACGACAGACTTT	qPCR primers
	CTGATACGTCAGAGCCGACC	qPCR primers
Lama2	CAGGCCCGAAACGCAAAC	qPCR primers
	GCTCACTGGCTGGTCGTT	qPCR primers
Lama4	TCGTATTGCCAGCTACCTGTTT	qPCR primers
	CAAAGTCGTACATTAGCCGCAG	qPCR primers
Lama5	ACGATGACAACAGCCACTACAT	qPCR primers
	TCTGAGTATCCGTTTCCCTCCT	qPCR primers
Lamb1a	AGCACTCTACGGCAACTCAC	qPCR primers
	ACACCGTCACATCCTTCACC	qPCR primers
Lamb1b	GGAGGCCGAGTTTCACTTCA	qPCR primers
	CTGAAAGACGGGGTCCAACA	qPCR primers
Lamb2l	CTGGGGAGTCACTGCAACAT	qPCR primers
	CGAGCACATTCATCACAGCG	qPCR primers
Lamb2	TCAACGGAGGGAGAGGTCAT	qPCR primers
	CCCAGAGTGTGAAGCTTGGT	qPCR primers
Lamb3	AGGACACCTCTAAGACTGGACCAAA	qPCR primers
	AAGCATACAGACGCAAAGGACTGAA	qPCR primers
Lamb4	GGTCCAAACTGTGACGGAGT	qPCR primers
	TGGAGGTCTTTGGCTTGGTC	qPCR primers
Lamc1	TACTTCTACAACCGCTCGGC	qPCR primers
	CGGTCTACGTCTTTGCTGGT	qPCR primers
Lamc2	CTGATGCAGGCGATGAAACG	qPCR primers
	AGAGCTTTGTTGGCGCTTTG	qPCR primers
Lamc3	TCATCAAGAAACAGGCGGAGAA	qPCR primers
	GAAGGCCAGAAAATCCTCCAGA	qPCR primers
Lama4	CCGAGGACTTCCAGCGATAC	ISH probe
	GCGCCGTCGTTGTATTTCTC	ISH probe
	TAATACGACTCACTATAGGGGCGCCGTCGTTGTATTTCTC	ISH probe
