# Supplementary figures and images for: Genome-Wide Identification of Laminin Family Related to Follicular Pseudoplacenta Development in Black Rockfish (Sebastes schlegelii)
Source: Int J Mol Sci. 2022 Sep 10;23(18):10523. doi: 10.3390/ijms231810523 (PMC9504374; doi:10.3390/ijms231810523)

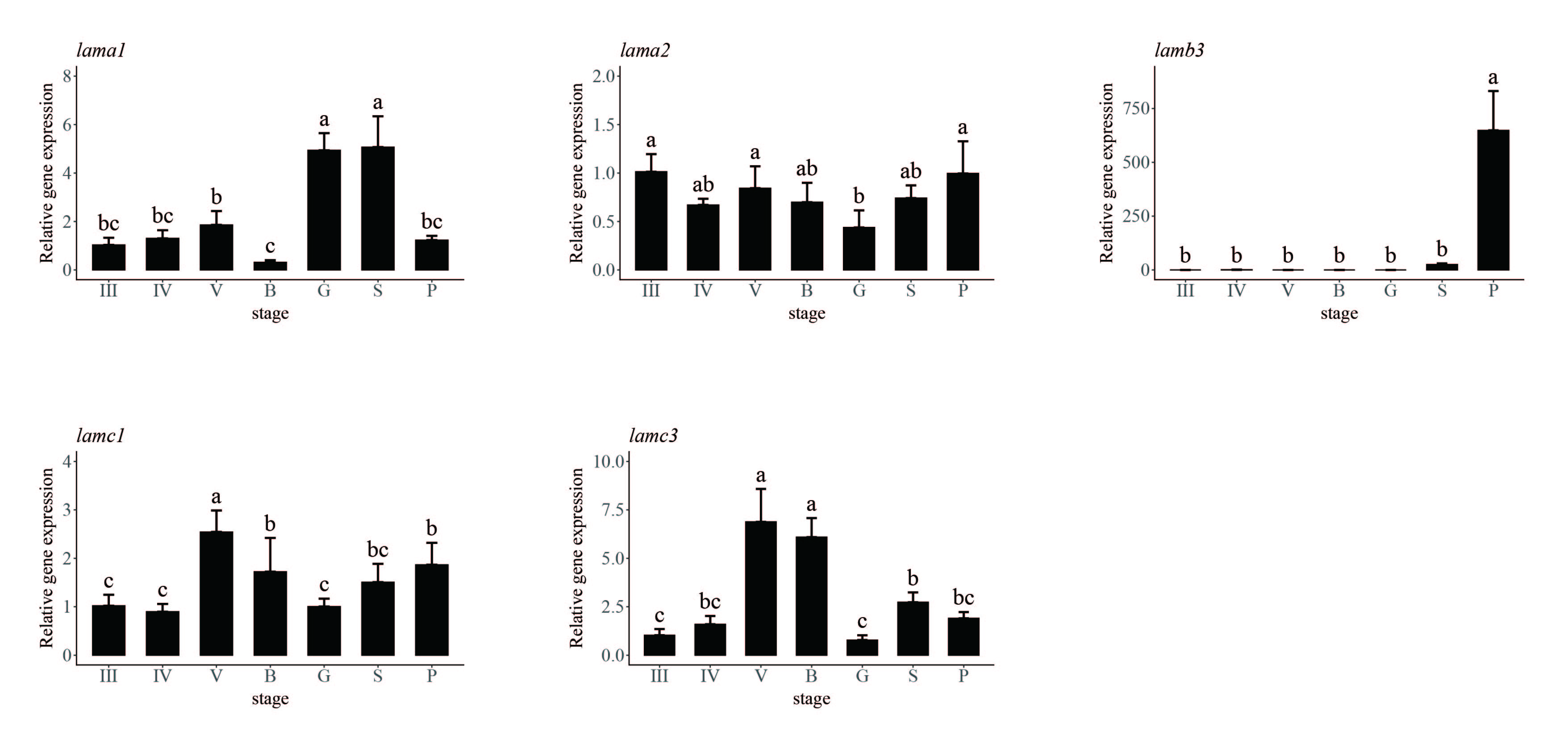

Supplement: Supplementary file 1 [file ijms-23-10523-s001.zip › Figure S1.jpg]
